# Simultaneous laparoscopic splenectomy and right hemihepatectomy for littoral cell angiosarcoma accompanied with liver metastases

**DOI:** 10.1186/1477-7819-11-215

**Published:** 2013-08-28

**Authors:** Liang Wang, Dianrong Xiu, Bin Jiang, Zhaolai Ma, Chunhui Yuan, Lei Li

**Affiliations:** 1 Department of General Surgery, Tsinghua University Beijng Tsinghua Hospital, Tiantongyuan, Changping District, Beijing 102218, China; 2 Department of General Surgery, Peking University Third Hospital, 49 North Garden Road, Haidian District, Beijing 100191, China

**Keywords:** Laparoscopic splenectomy, Laparoscopic right hemihepatectomy, Concomitant surgery, Littoral cell angiosarcoma

## Abstract

Despite the wide acceptance of laparoscopic resection for treatment of abdominal tumors, only few cases of simultaneous laparoscopic removal of the spleen and the right liver have been reported to date. Littoral cell angiosarcoma (LCAS), which arises from the littoral cells lining the sinus channels of the splenic red pulp, is a rare condition, and there is limited literature on littoral cell angiosarcoma with liver metastases. We present the case of a 28-year-old woman with postoperative pathologically-proven LCAS with right liver metastases. The patient’s surgery was safely performed, and her postoperative course was uneventful until now. This case suggests that concomitant laparoscopic splenectomy (LS) and right hemihepatectomy is a suitable surgical option for selected patients.

## Background

Both laparoscopic splenectomy (LS) and laparoscopic right hemihepatectomy (LRH) have been performed clinically, with the former being more widely accepted. Because of the advantages afforded by laparoscopic surgeries, simultaneous laparoscopic procedures have been performed for treating coexisting abdominal diseases/lesions [1]. Sasaki *et al*. [2] reported the findings for nine patients who safely underwent concomitant laparoscopic splenectomies and cholecystectomies. Ohno *et al*. [3] reported a case in which laparoscopic hand-assisted splenectomy and partial hepatectomy were performed simultaneously in a patient who showed liver cancer with hypersplenism. However, cases involving concomitant pure LS and right hemihepatectomy have not been reported so far.

We present a rare case of angiogenic tumors occurring in both the liver and the spleen in a 28-year-old woman. A preoperative biopsy of the patient’s spleen by immunohistochemical staining indicated that her tumors were angiogenic and that the possibility of littoral cell angioma (LCA), angiosarcoma, or littoral cell angiosarcoma (LCAS) could not be excluded. Moreover, computed tomography (CT) scans and magnetic resonance imaging (MRI) scans showed that the imaging features of the liver lesions were similar to those of the spleen. These image findings suggested that lesions in the liver and in the spleen might have the same origin. Therefore, we performed simultaneous LS and right hemihepatectomy.

## Case presentation

**Figure 1:**
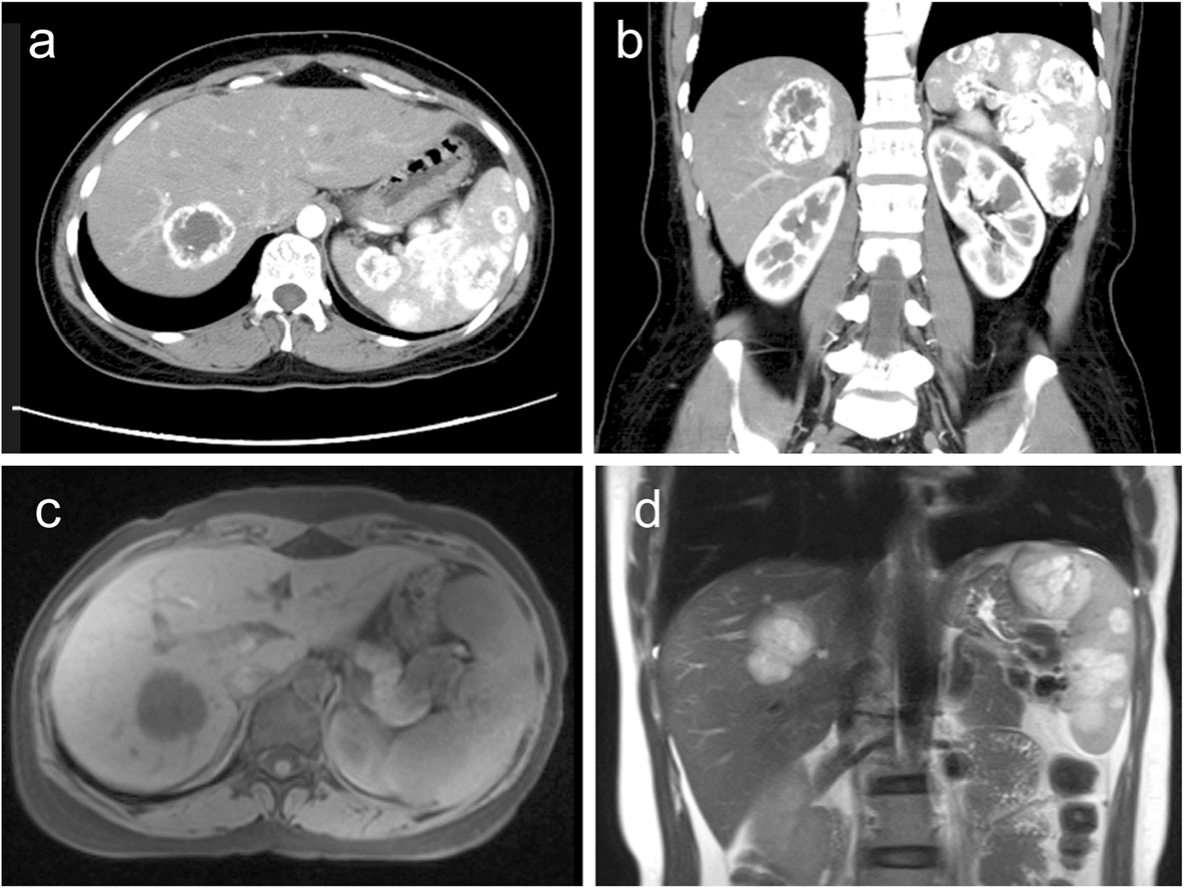
**Preoperative images of computed tomography (CT) and magnetic resonance imaging (MRI) scans.** Both the axial **(a)** and the coronal **(b)** CT images show multiple low-density nodules with enhanced nodule boundaries in the spleen and the liver. MR images of the splenic and hepatic masses show a low signal intensity on T1-weighted MR **(c)** images and a high signal intensity on T2-weighted MR **(d)** images.

A 28-year-old woman was referred to our department on 14 January 2011, because of a solid cystic mass in the spleen and the liver that had been incidentally identified by ultrasonography during a routine medical checkup. She had no complaints and symptoms. The patient’s physical examination showed no significant positive findings. Her laboratory data were within normal limits. Tests for the tumor markers CA19-9, CEA, and AFP yielded negative results. Ultrasonography showed the presence of solid nodules with liquefaction necrosis in the liver and the spleen. CT scan showed mild splenomegaly and multiple hypodense nodules with blurred boundaries in the spleen and the right liver. Contrast-enhanced CT showed enhanced nodule boundaries during the arterial phase and homogeneously enhanced nodules during the portal venous phase (Figure 1). On unenhanced MR images, the splenic and hepatic masses showed low signal intensity on T1-weighted MR images and a high signal intensity on T2-weighted MR images (Figure 1). The biopsy of spleen masses was done in a different hospital before she was referred to our department, and the biopsy showed hemangioma-like structures in a part of the tissue, no evidence of endothelial cell atypia, papillary projections in a part of the vessel lumen, and no abnormal mitosis in the cells. Biopsy specimens indicated a high possibility of LCA with positive expressions of CD34 and CD68.

### Surgical procedure

**Figure 2:**
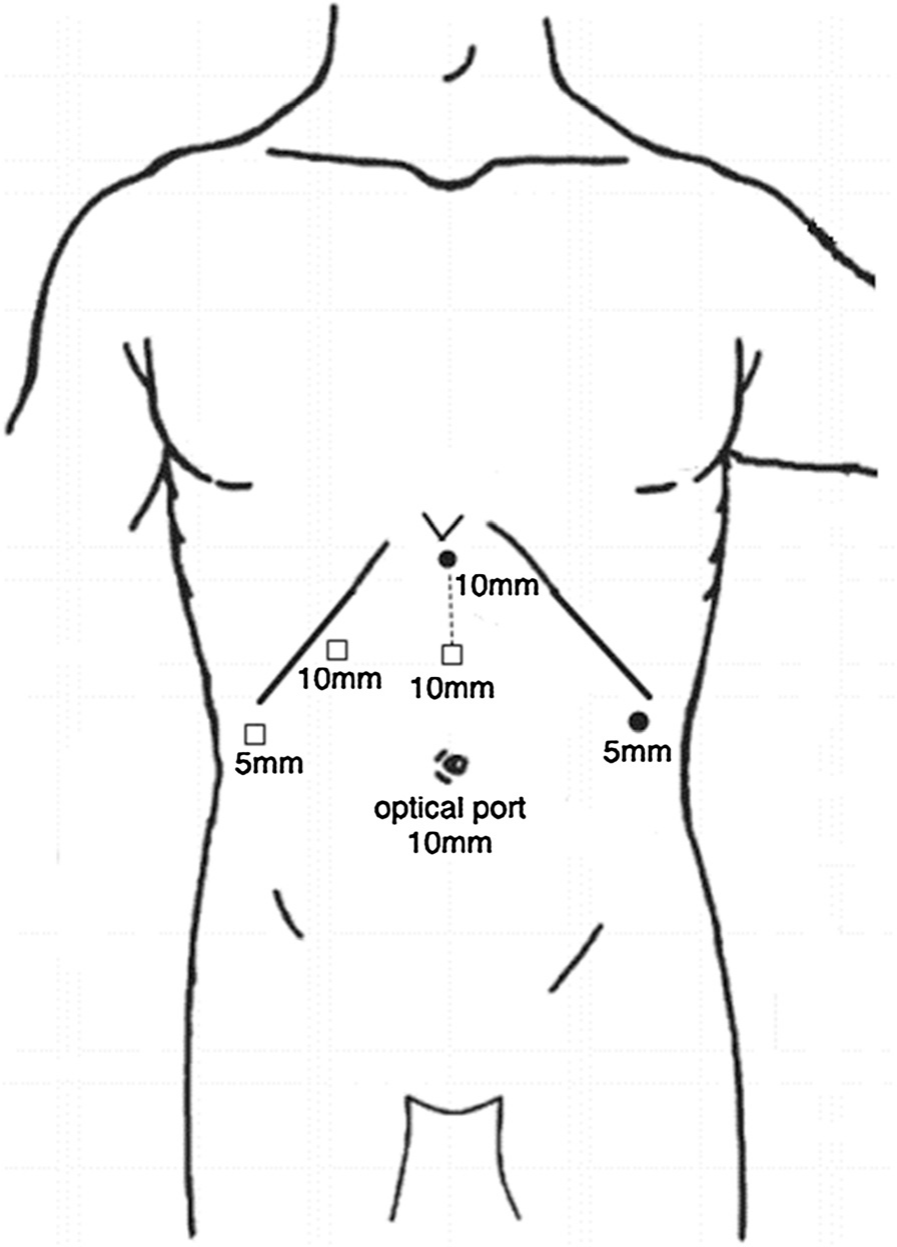
**Trocar placement.** ‘●’ stands for the trocar used in laparoscopic splenectomy (LS); ‘□’ standsstands for the trocar added for laparoscopic right hemihepatectomy (LRH); ‘……’ stands for the incision for specimen removal.

Surgery was performed under general anesthesia. Laparoscopy was performed under pneumoperitoneum with a maximum pressure of 10 mmHg carbon dioxide. A 30° laparoscope with a diameter of 10 mm was used to explore the abdominal cavity to ensure there was no diffused peritoneal dissemination. For LS, a 10-mm trocar and a 5-mm trocar acted as the working ports (Figure 2). The patient was then placed in the right semi-lateral and head-up tilt position. The spleen-colonic ligament was cut using an ultrasonic scalpel, after which the branches of the splenic artery and splenic vein were ligated and cut one by one. Finally, the spleen-gastric ligament and the spleen-renal ligament were cut using the ultrasonic scalpel.

**Figure 3:**
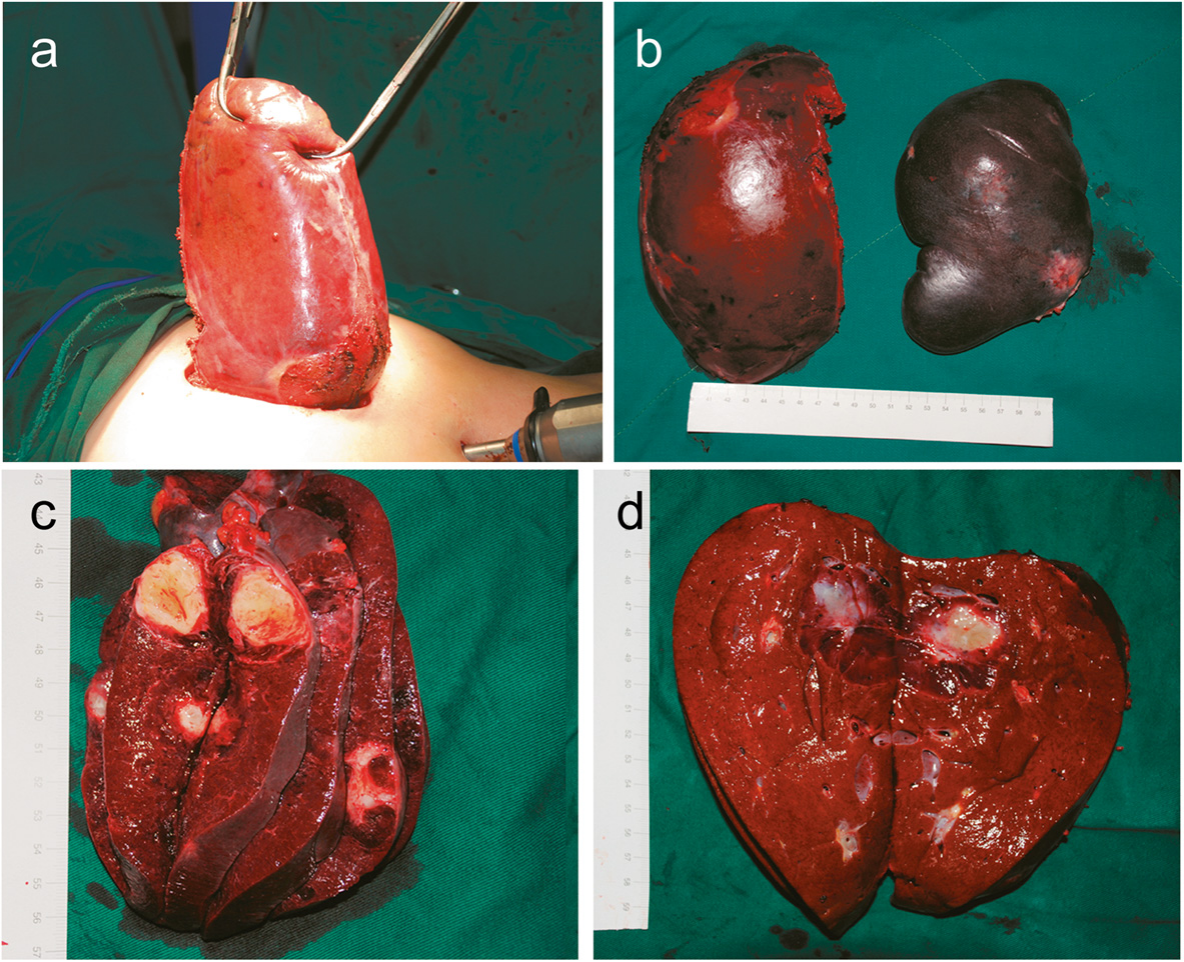
**Incision and specimens.** 8 cm-incision **(a)** and specimens **(b)**. The photo was taken after specimen extraction and the cleaning of the incision site. The cut section of the spleen **(c)** shows multiple white-gray or brownish-red nodules whose diameter ranged between 2.0 cm and 4.0 cm. The cut surface of the liver **(d)** showed a brownish-red nodule with a diameter of 4.0 cm.

After LS, the patient was adjusted in a left half-lateral and head-up tilt position and another 3 trocars were introduced for LRH, as shown in Figure 2. The falciform ligament was resected up to the roots of the hepatic vein by using an ultrasonic scalpel; the right lobe of the liver bed was divided from the right triangular and coronary ligaments until the adrenal gland; and the inferior vena cava were exposed. The right liver was mobilized by dividing the cystic artery and the duct of the gallbladder to efficiently expose the liver pedicle. The right hepatic pedicle, including the right hepatic artery, right hepatic portal vein, and right bile duct were transected through the extra-Glissonian approach [4,5] using linear staplers (45-mm Endo GIA; staple height, 2.5 mm; Tyco healthcare, Norwalk, CT, USA). Along the demarcation line, hepatic parenchyma transection was performed with ultrasound scalpels and Ligasure (Valley Lab, Boulder, CO, USA). Blood vessels larger than 5 mm in diameter were ligated with Hem-o-lok clips (Teleflex Medical, Research Triangle Park, NC, USA). The right liver was lifted anteriorly using two 5-mm graspers to create a tunnel. The posterior attachments of the liver were dissected to expose the retrohepatic inferior vena cava and the right hepatic vein. The right hepatic vein and the inferior right hepatic vein were divided using linear staplers. After the liver transection was finished, the gallbladder was dissected from its bed. An 8-cm midline incision was made between the two trocars (Figure 2). All tissue specimens were removed inside two plastic bags through this wound (Figure 3). Resection margins were carefully examined for bleeding and bile leaks, and abdominal drains were introduced in the spleen fossa and on the liver transection surface.

## Results

The operation lasted 482 min, and the estimated blood loss was 800 ml with no transfusion. Oral intake of food was restarted on the third postoperative day (POD), and the drains were removed on the sixth POD. The patient’s recovery was uneventful, and she was discharged from the hospital on the tenth day after the surgery.

**Figure 4:**
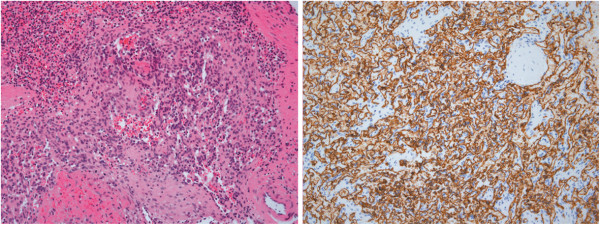
**Photomicrographs of the pathological specimen.** Hematoxylin-eosin staining showed that the lesions consisted of anastomosing vascular channels**.** Immunohistochemical stains showed positive immunoreactivity to CD34.

Postoperative gross pathologic examination showed mild enlargement of the spleen. The cut section of the spleen showed multiple white-gray or brownish-red nodules whose diameter ranged between 2.0 cm and 4.0 cm. The cut surface of the liver showed a brownish-red nodule with a diameter of 4.0 cm, whose histological structure was identical to that of the nodules in the spleen (Figure 3). Microscopically, the lesions consisted of anastomosing vascular channels. The cells lining the vascular spaces were tall, plump, and bland with few mitotic figures and no cytologic nuclear atypia (Figure 4). Immunohistochemical analysis of the tumors showed the following results: cells were positive for CD34, CD31, CK mix, CD68, PGM-1, CD8; they were negative for FVIII antigen, CD21 markers; and less than 2% of the cells were Ki-67-positive (Figure 4). The patient’s condition was diagnosed as spleen LCAS with liver metastases.

Follow-up CT scans obtained at 2 months, 4 months, and 1 year after surgery showed no signs of tumor recurrence.

## Discussion

In this case, the lesions in the right liver and the spleen were histopathologically angiogenic tumors that arose from the littoral cell. The LCA cells express both endothelial and histiocytic antigens, while the normal endothelia of the sinuses express only endothelial antigens [6]. Therefore, the immunophenotypic signature of these cells, that is, expression of CD31, CD68, CD163, FVIII antigen, and CD21 markers on the lining of the cells, was considered unique to LCA. Usually, the cells are negative for CD34 and CD8 in LCA. Unlike LCA, the tumor cells in this case were negative for CD21 and FVIII antigen and positive for CD34 and CD8. Rosso *et al*. [7-9] reported a case of LCAS with positive expression of CD34 and CD8 in cells and a poor clinical prognosis. Arber and Ben-Izhak *et al*. [10,11] reported that LCAS can be differentiated from LCA and indolent littoral cell hemangioendothelioma. However, the significance of CD21 expression in LCA or LCAS is not known yet [10]. Thus, to determine the role of CD21 expression in LCA, the patient in this case needs long-term follow-up.

Minimally invasive surgery has been accepted worldwide. LS has become the standard approach for most splenectomy cases [12,13]. Though there are no randomized controlled trial results that support laparoscopic right hepatectomy for treatment of liver tumors, a consensus over laparoscopic liver resection was reached in 2009, providing evidence that laparoscopic right hepatectomy can be performed by experienced surgeons [14].

Conventional open surgical procedures for tumor resection would require a very big incision since the spleen and right liver are located at the opposite positions. Laparoscopic surgery has many advantages over other open surgical procedures, such as less postoperative pain, reduced wound complications, a quick recovery, reduced surgical stress, and a potentially lower probability of recurrence of malignant tumors. Recent innovations in laparoscopic instruments and surgical techniques make routine resections of solid organs clinically possible. However, the laparoscopic approach has been rarely applied to simultaneous resections of the right liver and the spleen owing to its presumed technical difficulty and concerns about massive intraoperative bleeding.

In this case, a total LS, right hemihepatectomy, and cholecystectomy were safely accomplished by an expert surgeon. The patient’s position was changed by adjusting the operation table. Thus, LRH could be performed after LS without moving the patient. Reducing intraoperative blood loss is the key factor in ensuring surgical success. Limiting blood inflow through the liver by right hepatic pedicle transection can create a bloodless operating field, markedly reducing intraoperative blood loss and avoiding ischemia-reperfusion injury to the liver with the Pringle maneuver technique. Transection of liver parenchyma along the ischemic demarcation line revealed few liver inflow vessels. By lowering the central venous pressure, bleeding from the hepatic vein was markedly reduced [15]. The right hepatic vein was transected after the transection of the liver parenchyma as this method exposed the vein more clearly. The combined use of ultrasonic scalpels, LigaSure, and Hem-o-lok was safe and highly effective as far as hemostasis was concerned [16]. In addition, the laparoscopic approach alleviated the young female patient’s concern about her outward appearance. Tisdale *et al*. [17], Teixeira *et al*. [18] and deSouza *et al*. [19] reported that a Pfannenstiel incision can be preferable to a midline incision in terms of cosmetic concerns, efficiency, or incisional hernia rate reduction when dealing with large surgical specimens. A midline incision was indeed used in our procedure and Pfannenstiel incisions might be attempted in suitable future cases.

## Conclusions

In conclusion, in rare cases of LCAS with synchronous liver metastasis, in which proper hemostasis and surgeries are performed by experienced surgeons, concomitant LS and right hemihepatectomy are a viable treatment option in selected patients.

## Consent

Written informed consent was obtained from the patient for the publication of this report and any accompanying images.

## Abbreviations

CT: Computed tomography; LCA: Littoral cell angioma; LCAS: Littoral cell angiosarcoma; LRH: Laparoscopic right hemihepatectomy; LS: Laparoscopic splenectomy; MRI: Magenetic resonance imaging; POD: Postoperative day.

## Competing interests

The authors declare that they have no competing interests.

## Authors’ contributions

L Wang wrote this case report. D Xiu was the surgeon, Z Ma, B Jiang, L Li and L Wang were assistants. C Yuan provided the photos and artworks in this case report. All authors read and approved the final manuscript.
